# Comparison of disease and economic burden between MRSA infection and MRSA colonization in a university hospital: a retrospective data integration study

**DOI:** 10.1186/s13756-024-01383-8

**Published:** 2024-02-29

**Authors:** Aki Hirabayashi, Koji Yahara, Keisuke Oka, Toshiki Kajihara, Teruko Ohkura, Yumiko Hosaka, Keigo Shibayama, Motoyuki Sugai, Tetsuya Yagi

**Affiliations:** 1 Antimicrobial Resistance Research Center, National Institute of Infectious Diseases, Tokyo, Japan; 2 Department of Infectious Diseases, Nagoya University Hospital, Aichi, Japan; 3 Department of Medical Technique, Nagoya University Hospital, Aichi, Japan; 4 Department of Bacteriology, Nagoya University Graduate School of Medicine, Aichi , Japan

**Keywords:** MRSA infections, MSSA infections, MRSA colonization, In-hospital mortality, Length of stay, Hospital charges

## Abstract

**Background:**

Although there is a growing concern and policy regarding infections or colonization caused by resistant bacteria, including methicillin-resistant *Staphylococcus aureus* (MRSA), the prognosis of MRSA infections compared to that of methicillin-susceptible *Staphylococcus aureus* (MSSA) infections remains controversial. Moreover, there have not been any studies comparing both the burden of disease and its impact on the healthcare economy between MRSA infection and colonization while adjusting for confounding factors. These comparisons are crucial for developing effective infection control measures and healthcare policies. We aimed to compare the disease and economic burden between MRSA and MSSA infections and between MRSA infection and colonization.

**Methods:**

We retrospectively investigated data of 496 in-patients with MRSA or MSSA infections and of 1178 in-patients with MRSA infections or MRSA colonization from a university hospital in Japan from 2016 to 2021. We compared in-hospital mortality, length of stay, and hospital charges between in-patients with MRSA and MSSA infections and those with MRSA infections and MRSA colonization using multiple regressions. We combined surveillance data, including all microbiological test results, data on patients with infections, treatment histories, and clinical outcomes, to create the datasets.

**Results:**

There was no statistically significant difference in in-hospital mortality rates between matched MRSA vs. MSSA infections and MRSA infection vs. colonization. On the contrary, the adjusted effects of the MRSA infection compared to those of MSSA infection on length of stay and hospital charges were 1.21-fold (95% confidence interval [CI] 1.03–1.42, *P* = 0.019) and 1.70-fold (95% CI 1.39–2.07, *P* < 0.00001), respectively. The adjusted effects of the MRSA infection compared to those of MRSA colonization on length of stay and hospital charges were 1.41-fold (95% CI 1.25–1.58, *P* < 0.00001) and 1.53-fold (95% CI 1.33–1.75, *P* < 0.00001), respectively. Regarding confounding factors, hemodialysis or hemofiltration was consistently identified and adjusted for in the multiple regression analyses comparing MRSA and MSSA infections, as well as MRSA infection and MRSA colonization.

**Conclusions:**

MRSA infection was associated with longer length of stay and higher hospital charges than both MSSA infection and MRSA colonization. Furthermore, hemodialysis or hemofiltration was identified as a common underlying factor contributing to increased length of stay and hospital charges.

**Supplementary Information:**

The online version contains supplementary material available at 10.1186/s13756-024-01383-8.

## Background

Worldwide, the number of bacteria resistant to antimicrobials is rising; as a result, nations are implementing national action plans combating antimicrobial resistance and other strategies such as guidance to fight infections. Infection caused by resistant bacteria has limited treatment options and can be severe, requiring substantial medical resources; furthermore, quantitatively assessing its impact on severity or mortality and healthcare economics is extremely important for developing more effective infection control measures and healthcare policies in the future. Among the resistant bacteria, methicillin-resistant *Staphylococcus aureus* (MRSA) is often endemic in a state of asymptomatic carriage, known as colonization, even in healthy people without immunosuppression. MRSA colonization is one of the risk factors for MRSA infection [1, 2]; however, comparing MRSA colonization to MRSA infection in terms of severity, mortality, and healthcare economics is challenging as it requires a professional diagnosis to differentiate between the two conditions for a sufficient number of patients.

MRSA infections have a worse prognosis and higher hospital charges than methicillin-susceptible *Staphylococcus aureus* (MSSA) infections do [3, 4]. A meta-analysis analyzing the studies on the impact of methicillin-resistance on mortality in *Staphylococcus aureus* bacteremia from 1980 to 2000 showed a significant increase in mortality associated with MRSA bacteremia (OR, 1.93; 95% CI, 1.54–2.42; *P* < 0.001) [4]. In another systematic review, methicillin resistance was associated with high mortality in ventilator-associated pneumonia caused by *S. aureus*, although certain confounders, like the adequacy of the empirical treatment and severity of illness, may be present and could contribute to the association [5]. Regarding medical costs, several studies have reported MRSA infections to be 2–3 times more expensive than MSSA infections [6, 7]. In Japan, Uematsu et al. reported that length of stay, hospital charges, and mortality were 1.03 (95% CI, 1.01–1.05), 1.04 (95% CI, 1.01–1.06), and 1.14 (95% CI, 1.02–1.27) times higher in the MRSA group than in the MSSA group in a comparison between MRSA and MSSA infections from 2014 to 2016 [8]. In another study from a single institution in Japan performed from 2016 to 2020, both healthcare costs and mortality rates were relatively high in the MRSA bacteremia group [9]. Regarding MRSA colonization, adjusted mortality rates in patients with infection were almost twice as high compared to those in the patients who had only nasal colonization [10]. Additionally, a few studies have focused on the burden of MRSA colonization on healthcare costs [11, 12].

However, to our knowledge, no study has compared the overall burden of mortality, length of stay, and hospital charges associated with MRSA infection and colonization while adjusting for confounding factors. Objective data are needed to determine the level of caution that should be exercised in the state of colonization. In this study, we focused on Japan, which has a high prevalence of MRSA (46% in Japan vs. 17.7% in EU/EEA) and a national comprehensive antimicrobial resistance surveillance program, the Japan Nosocomial Infections Surveillance (JANIS) [13, 14]. Since 2000, the Clinical Laboratory (CL) division of JANIS has collected routine microbiological test results for all sample types from both symptomatic and asymptomatic patients in hospitals [15]. The number of participating hospitals exceeded 2,800 as of January 2023. The national surveillance data from 2021 showed that MRSA was isolated from 167,858 patients (6.02% of the total number of specimen-submitting patients), which was the highest among antimicrobial-resistant bacteria under surveillance [13]. The Antimicrobial-Resistant Bacterial Infection (ARBI) division of JANIS collects data from only symptomatic patients with seven major antimicrobial-resistant bacteria, including MRSA, based on professional diagnoses done by medical doctors as part of routine medical practice. National data on symptomatic patients in 2021 showed that the incidence rate of MRSA infections was 2.79‰ of the total number of hospitalized patients, which was also the highest among antimicrobial-resistant bacteria under surveillance. Combining the two types of data from JANIS will make it possible to distinguish between MRSA infection and colonization, as well as the infection caused by MSSA, at the individual patient level. In addition, large Japanese hospitals usually collect data on the diagnosis, comorbidities, treatment history, hospital charges, and clinical outcomes at the discharge of each patient and regularly submit them to the Ministry of Health, Labor and Welfare in Japan, as the Japanese Diagnosis Procedure Combination (DPC) administrative claims the database [16, 17]. The DPC data and the two types of JANIS data are typically stored in separate databases within Japanese hospitals, and combining these datasets will enable a comparison of the disease and economic burden between MRSA and MSSA infections, as well as MRSA colonization. Integrating such data poses a cutting-edge challenge at a global level, as exemplified in projects like the Global Research on Antimicrobial Resistance Project [18].

Based on the data integration, we aimed to compare the in-hospital mortality, total length of stay, and total hospital charges between MRSA and MSSA infections and between MRSA infection and colonization. Our study involved data integration and comparison over six years at a university hospital with a total of 1080 beds, which participated in JANIS and stored the data in the DPC database. Our study provided a detailed understanding of the disease burden and associated hospital charges through two types of comparisons stratified by infection and colonization. This holds significance not only for infection control but also for healthcare economics.

## Methods

This retrospective study compared the disease and economic burden between MRSA and MSSA infections and between MRSA infection and colonization. This study utilized data integration from Nagoya University Hospital, which participates in JANIS. Among the five divisions of JANIS (CL, ARBI, intensive care unit (ICU), surgical site infection, and neonatal intensive care unit (NICU)), Nagoya University Hospital has been participating in the CL and ARBI divisions. The CL division collects comprehensive specimen-based microbiology data, regardless of whether infection or colonization occurs, in the diagnostic microbiology laboratories of the participating hospitals. The ARBI division collects data from patients with infections caused by specific resistant organisms, including MRSA. The medical doctors made diagnoses of infections as part of their routine practices, which were recorded in the database of ARBI division of JANIS. By integrating the microbiology and patient data from both divisions, it was possible to distinguish between MRSA infections and colonization. In addition, DPC data included clinical information, treatment history, hospital charges, and outcomes of the patients. Each dataset is stored under different anonymized identifications (IDs) for personal information protection. The three datasets are typically stored in separate databases within Japanese hospitals including Nagoya University Hospital. We combined these datasets for each patient using an in-house Perl program that incorporated a table displaying the correspondence between the previously anonymized IDs and the new anonymous IDs generated from the original patient IDs, as described in detail below.

From the data of the ARBI division, patients diagnosed with MRSA infections were selected and matched 1:1 with MSSA infection patients with the same specimen type and the type of the infection from the DPC data, as shown in Table S1. In a subgroup analysis that focused on patients with bacteremia, only patients with positive blood cultures from each group were selected for comparison between the MRSA bacteremia group and the MSSA bacteremia group. For the colonization group, we selected patients who were not on the list of MRSA infections from the ARBI division but who had specimens of detected MRSA from the CL division. The following data were extracted from the DPC data for each patient: age, sex, Charlson Comorbidity Index, ICU admission, mechanical ventilation, hemodialysis (HD) or hemofiltration (HF), in-hospital mortality, total length of stay, and total hospital charges. The Charlson Comorbidity Index was used to assess the patients’ backgrounds. To convert the ICD-10 codes included in the DPC to the Charlson Comorbidity Index, ICD-10 coding algorithms for Charlson comorbidities were used, according to a previous study [19]. The data for these variables were collected both before and after the onset of *S. aureus* infection. Hence, this study was not intended to estimate causal relationships between individual variables such as HD or HF and the development of infection. Rather, the study aimed to conduct between-group comparisons regarding in-hospital mortality, length of stay, and hospital charges as follows.

Using the combined dataset, we performed three between-group comparisons. In each comparison (MRSA vs. MSSA infection, MRSA vs. MSSA bacteremia, and MRSA infection vs. colonization), we first performed a univariate analysis to examine the association between the group and clinical variables of patients using Fisher’s exact test for discrete variables and Wilcoxon’s rank-sum test for continuous variables. For each clinical variable with *p*-values less than 0.1 according to the univariate analysis, we explored confounding factors that were also associated with in-hospital mortality, length of stay, and total hospital charges (with *p*-values less than 0.1), using negative-binomial regression, linear regression in which the outcome variable was log-transformed, and Cox regression. We used negative-binomial regression and linear regression in which the outcome variable was log-transformed due to long-tail distributions of length of stay and total hospital charges. Subsequently, multiple Cox regression, negative binomial regression, and linear regression were performed to obtain the *p*-value for the association between the patient group and in-hospital mortality, length of stay, and total hospital charges, respectively, after adjusting for the confounding factors. If the confounding factors were highly associated (*p* < 0.001 determined using Wilcoxon’s rank sum test or Fisher’s exact test) with each other, we selected one of them as a representative to avoid multicollinearity based on medical knowledge and included it in the multiple regression analysis as a covariate to be adjusted for. Statistical analyses were performed using the R software (version 4.1.2) and JMP Pro version 13 (SAS Institute, Cary, NC, USA).

## Results

### MRSA infection vs. MSSA infection

Comparison of clinical characteristics between patients with MRSA infections and those with MSSA infections, matched by specimen type and diagnosis (Table 1), revealed differences in the univariate analysis at a significance level of 0.1 for the following explanatory variables: C3_Peripheral vascular disease (*P* = 0.09), C12_Renal disease (*P* = 0.04), ICU admission (*P* = 0.0003), mechanical ventilation (*P* < 0.00001), and HD or HF (*P* = 0.0009); all of these were more prevalent in the MRSA infection group than in the MSSA infection group. The data for these potentially risky variables were collected both before and after the onset of *S. aureus* infection. As outlined in the Methods section, the primary aim of this study was not to establish causal relationships between individual variables and the development of infection. Instead, the listed variables were scrutinized as potential confounding factors, as detailed in the subsequent paragraph. There were also significant differences in length of stay (median, 44 vs. 34 days, *P* = 0.0001) and hospital charges (median, $27,215 vs. $12,525, *P* < 0.00001). However, no significant difference was found in the in-hospital mortality.

**Table 1:** Characteristics of the patients with MRSA and MSSA infections matched based on specimen type and diagnosis

Variables	MRSA infection n (%)	MSSA infection n (%)	*P* value
Number of patients	248	248	
Age, median (IQR), years	69 (49,76)	68 (51,78)	n.s.
Male sex, *n* (%)	166 (68.0%)	152 (61.0%)	n.s.
Categories of summation of the Charlson Comorbidity Index			
0, indicating low	73 (29.0%)	65 (26.0%)	n.s.
1 or 2, indicating medium	103 (42.0%)	117 (47.0%)	n.s.
3 or 4, indicating high	54 (22.0%)	51 (21.0%)	n.s.
≥ 5, indicating very high	18 (7.3%)	15 (6.0%)	n.s.
Charlson Comorbidity Index			
C1_Myocardial infarction	3 (1.2%)	8 (3.2%)	n.s.
C2_Congestive heart failure	35 (14.0%)	35 (14.0%)	n.s.
C3_Peripheral vascular disease	35 (14.0%)	22 (8.9%)	0.09
C4_Cerebrovascular disease	12 (4.8%)	17 (6.9%)	n.s.
C5_Dementia	5 (2.0%)	7 (2.8%)	n.s.
C6_Chronic pulmonary disease	14 (5.6%)	12 (4.8%)	n.s.
C7_Rheumatic disease	8 (3.2%)	10 (4.0%)	n.s.
C8_Peptic ulcer disease	11 (4.4%)	16 (6.5%)	n.s.
C9_Mild liver disease	8 (3.2%)	13 (5.2%)	n.s.
C10_Diabetes without chronic complication	31 (13.0%)	37 (15.0%)	n.s.
C11_Hemiplegia or paraplegia	2 (0.8%)	0 (0%)	n.s.
C12_Renal disease	34 (14.0%)	19 (7.7%)	0.04
C13_Diabetes with chronic complication	15 (6.0%)	10 (4.0%)	n.s.
C14_Malignancy	65 (26.0%)	67 (27.0%)	n.s.
C15_Leukemia	5 (2.0%)	4 (1.6%)	n.s.
C16_Lymphoma	5 (2.0%)	2 (0.8%)	n.s.
C17_Moderate or severe liver disease	2 (0.8%)	5 (2.0%)	n.s.
C18_Metastatic solid tumor	8 (3.2%)	9 (3.6%)	n.s.
C19_AIDS/HIV	0 (0%)	0 (0%)	n.s.
ICU admission	130 (52.0%)	89 (36.0%)	0.0003
Mechanical ventilation	98 (40.0%)	49 (20.0%)	< 0.00001
HD or HF	31 (13.0%)	10 (4.0%)	0.0009
In-hospital mortality	36 (15.0%)	26 (10.0%)	n.s.
Length of stay, median (IQR), days	44 (26, 81)	34 (15, 61)	0.0001
Hospital charges, median (IQR), US dollars	27,215 (10,038, 65,809)	12,525 (5,722, 26,201)	< 0.00001

Abbreviation: IQR, interquartile range

n.s.: *P*$$ \geqslant $$ 0.1

Next, multiple regression analyses adjusted for confounding factors were performed for length of stay and hospital charges, showing a significant difference in the univariate analysis. Owing to the long-tailed distribution of length of stay and hospital charges (Figure S1 and Figure S2 for the MRSA and MSSA infection groups, respectively), we employed negative binomial regression and linear regression in which the hospital charges were log-transformed. Regarding the length of stay, the confounding factors were C3_Peripheral vascular disease, ICU admission, mechanical ventilation, and HD or HF (Table S2). Based on the examination of pairwise associations among the confounding factors (Table S3), we selected C3_Peripheral vascular disease and HD or HF as representatives of those that were highly associated (*P* < 0.001) with each other. After adjusting for them, the effect of MRSA infection compared to that of MSSA infection on length of stay was estimated to be 1.21-fold (95% confidence interval [CI] 1.03–1.42, *P* = 0.019) (Table 2).

**Table 2:** Estimated effect of MRSA infection compared to that of MSSA infection on length of stay and hospital charges

Response variables	Explanatory variables	Ratio	95% CI	*P* value
Length of stay	MRSA infections vs. MSSA infections	1.21	1.03–1.42	0.019
	C3__ Peripheral vascular disease	1.14	0.89–1.46	0.031
	HD or HF	2.08	1.56–2.77	< 0.00001
Hospital charges	MRSA infections vs. MSSA infections	1.70	1.39–2.07	< 0.00001
	C3__ Peripheral vascular disease	1.29	0.94–1.76	0.111
	HD or HF	5.13	3.56–7.41	< 0.00001

As for the total hospital charges, the confounding factors were C3_Peripheral vascular disease, C12_renal disease, ICU admission, mechanical ventilation, and HD or HF (Table S2). Based on the examination of pairwise associations among the confounding factors (Table S3), we selected C3_Peripheral vascular disease and HD or HF as representatives of those that were highly associated (*P* < 0.001) with each other. After adjusting for them, the effect of MRSA infection compared to that of MSSA infection was estimated to be 1.70-fold (95% CI 1.39–2.07, *P* < 0.00001), as shown in Table 2.

According to the multiple regression model, the estimated increase in hospital charges for MRSA infection patients with peripheral vascular disease and HD or HF compared to those for MSSA infection patients with the same conditions was $59,291 (per patient, measured in dollars). The estimated increase in hospital charges for MRSA infection patients without peripheral vascular disease and HD or HF, compared to those for MSSA infection patients with the same conditions, was $8,058.

Further analyses focusing on the patients with bacteremia showed consistent results, as described in the Supplementary Text.

### MRSA infection vs. MRSA colonization

Comparison of clinical characteristics between all patients with MRSA infections and those with MRSA colonization (Table 3) revealed differences in the univariate analysis at significant level of 0.1 for the following explanatory variables: age (*P* = 0.0002), summation of Charlson Comorbidity Index indicating "high" (*P* = 0.01), C3_Peripheral vascular disease (*P* = 0.004), C4_Cerebrovascular disease (*P* = 0.01), C12_Renal disease (*P* = 0.002), C13_Diabetes with chronic complication (*P* = 0.07), and HD or HF (*P* = 0.01). The median age of the MRSA colonization group was lower than that of the MRSA infection group, with the lower bound of the interquartile range (IQR) being one year. Out of the 758 patients in the MRSA colonization group, 182 were 0 years old, and among these infants, 75.8% (138 out of 182) were admitted to the NICU. The categorical explanatory variables were more prevalent in the MRSA infection group, except for C4_Cerebrovascular disease, than in the MRSA colonization group. The comparison also revealed significant differences in in-hospital mortality (11.0% vs. 6.9%, *P* = 0.02), length of stay (median, 43 vs. 27 days, *P* < 0.00001), and hospital charges (median, $24,733 vs. $15,952, *P* < 0.00001).

**Table 3:** Characteristics of the patients with MRSA infection and MRSA colonization

Variables	MRSA infection n (%)	MRSA colonization n (%)	*P* value
Number of patients	420	758	
Age, median (IQR), years	68 (49, 76)	61 (1, 75)	0.0002
Male sex, *n* (%)	262 (64.0%)	467 (63.0%)	n.s.
Categories of summation of the Charlson Comorbidity Index			
0, indicating low	131 (31.0%)	263 (35.0%)	n.s.
1 or 2, indicating medium	166 (40.0%)	329 (43.0%)	n.s.
3 or 4, indicating high	95 (23.0%)	125 (16.0%)	0.01
≥ 5, indicating very high	28 (6.7%)	41 (5.4%)	n.s.
Charlson Comorbidity Index			
C1_Myocardial infarction	4 (1.0%)	16 (2.1%)	n.s.
C2_Congestive heart failure	50 (12.0%)	104 (14.0%)	n.s.
C3_Peripheral vascular disease	52 (12.0%)	54 (7.1%)	0.004
C4_Cerebrovascular disease	17 (4.0%)	60 (7.9%)	0.01
C5_Dementia	7 (1.7%)	17 (2.2%)	n.s.
C6_Chronic pulmonary disease	21 (5.0%)	37 (4.9%)	n.s.
C7_Rheumatic disease	11 (2.6%)	14 (1.8%)	n.s.
C8_Peptic ulcer disease	16 (3.8%)	32 (4.2%)	n.s.
C9_Mild liver disease	19 (4.5%)	22 (2.9%)	n.s.
C10_Diabetes without chronic complication	57 (14.0%)	81 (11.0%)	n.s.
C11_Hemiplegia or paraplegia	2 (0.5%)	3 (0.4%)	n.s.
C12_Renal disease	52 (12.0%)	51 (6.7%)	0.002
C13_Diabetes with chronic complication	21 (5.0%)	21 (2.8%)	0.07
C14_Malignancy	121 (29.0%)	215 (28.0%)	n.s.
C15_Leukemia	6 (1.4%)	10 (1.3%)	n.s.
C16_Lymphoma	4 (1.0%)	9 (1.2%)	n.s.
C17_Moderate or severe liver disease	4 (1.0%)	6 (0.8%)	n.s.
C18_Metastatic solid tumor	16 (3.8%)	26 (3.4%)	n.s.
C19_AIDS/HIV	0 (0%)	1 (0.1%)	n.s.
ICU admission	210 (50.0%)	394 (52.0%)	n.s.
Mechanical ventilation	143 (34.0%)	287 (38.0%)	n.s.
HD or HF	43 (10.0%)	46 (6.1%)	0.01
In-hospital mortality	46 (11.0%)	52 (6.9%)	0.02
Length of stay, median (IQR), days	43 (24, 77)	27 (14, 52)	< 0.00001
Hospital charges, median (IQR), US dollars	$ 24,733 (11,136, 56,484)	$ 15,952 (6,880, 35,603)	< 0.00001

Next, multiple regression analyses, adjusted for confounding factors, were performed for the three response variables that showed significant differences in the univariate analysis. Owing to the long-tailed distributions of length of stay and hospital charges (Figure S3 and S4 for the MRSA infection and colonization groups, respectively), we employed negative binomial regression and linear regression in which the hospital charges were log-transformed. As for length of stay, confounding factors were age, summation of Charlson Comorbidity Index indicating “high,” C3_Peripheral vascular disease, C4_Cerebrovascular disease, C12_Renal disease, and HD or HF (Table S4). Based on the examination of pairwise associations among the confounding factors (Table S5), we selected age and HD or HF as representatives of those that were highly associated (*P* < 0.001) with each other. After adjusting for them, the effect of MRSA infection compared to that of MRSA colonization on length of stay was estimated to be 1.41-fold (95% CI 1.25–1.58, *P* < 0.00001) (Table 4). As for the total hospital charges, confounding factors were age, C3_Peripheral vascular disease, C12_Renal disease, C13_Diabetes with chronic complications, and HD or HF (Table S4). Based on the examination of pairwise associations among the confounding factors (Table S5), we selected age and HD or HF as representatives of those that were highly associated (*P* < 0.001) with each other. After adjusting for them, the effect of MRSA infection compared to that of MRSA colonization was estimated to be 1.53-fold (95% CI 1.33–1.75, *P* < 0.00001), as shown in Table 4. According to the multiple regression model, the estimated increase in hospital charges for patients with MRSA infection, in contrast to those with MRSA colonization, was $44,146 (measured in dollars), considering an average age of 51 years (representative of all MRSA infection and MRSA colonization patients) and presence of HD or HF. On the other hand, the estimated increase was $7,076 for the same age group without HD or HF. By contrast, the difference in in-hospital mortality was not significant after adjusting for the confounding factors. When patients in the NICU were excluded from the analysis, the results remained unchanged.

**Table 4:** Estimated effect of MRSA infection compared to that of MRSA colonization on in-hospital morality, length of stay, and hospital charges

Response variables	Explanatory variables	Ratio	95% CI	*P* value
In-hospital mortality	MRSA infections vs. colonization	0.88	0.56–1.36	0.562
	age	1.13^#^	1.07–1.19^#^	< 0.00001
	HD or HF	2.04	1.23–3.38	0.005
Length of stay	MRSA infections vs. colonization	1.41	1.25–1.58	< 0.00001
	age	0.97^#^	0.96–0.98^#^	< 0.00001
	HD or HF	2.79	2.26–3.44	< 0.00001
Hospital charges	MRSA infections vs. colonization	1.53	1.33–1.75	< 0.00001
	age	0.97^#^	0.96–0.98^#^	< 0.00001
	HD or HF	6.24	4.89–7.95	< 0.00001

^#^ per 5 years old

## Discussion

To the best of our knowledge, in this study, we integrated three types of datasets for the first time to compare the disease and economic burden between MRSA and MSSA infections and between MRSA infection and colonization. Data integration has not yet been performed automatically; it can only be performed by first preparing raw data files and a table displaying the correspondence between the previously anonymized IDs and new anonymous IDs generated from the original patient IDs stored in each participating hospital in the surveillance.

Regarding in-hospital mortality, no significant difference was observed between MRSA and MSSA infection or bacteremia and between MRSA infection and colonization. Notably, this result is not consistent with the findings of a previous review in 2007 [3] and a previous study conducted in Japan from 2014 to 2016 [8]; both the studies reported that MRSA bacteremia nearly doubled the risk of mortality, compared with MSSA bacteremia. Delayed initiation of appropriate antimicrobial therapy for MRSA can result in increased fatality rates; however, this does not necessarily indicate that MRSA is inherently more virulent than MSSA [4, 20]. According to a previous study, in one institution, 32.6% of MRSA bacteremia were treated with inappropriate antimicrobials, and a statistically significant relationship was observed between the rates of inadequate antimicrobial treatment and the rates of in-hospital mortality [21]. Conversely, our findings suggest that the diagnosis and treatment, along with infectious disease consultations and interventions, may have been successful during the study period in this university hospital, where infectious disease doctors intervened in the treatment of all blood culture-positive patients and provided consultation in many infectious disease practices, including the ICU and other severely ill patients and those with antimicrobial-resistant bacteria. This is consistent with the findings of recent studies, which showed that the diagnosis and treatment of *S. aureus* have improved with the introduction of new antimicrobials and increased opportunities for infectious disease consultations to manage infections at the right time [22, 23].

According to a meta-analysis [4], 24 studies (77.4%) found no significant difference in the mortality rates for MRSA and MSSA bacteremia, 7 studies (22.6%) found significantly higher mortality rates for MRSA bacteremia relative to those of MSSA bacteremia, and no studies found significantly lower mortality rates for MRSA bacteremia relative to those of MSSA bacteremia. Meanwhile, an association between mortality and specific virulent genes of *S. aureus* has not been proven [24]. Although some studies have indicated that specific staphylococcal cassette chromosome *mec* (SCC*mec*) types are associated with high mortality [24–26], the association between SCC*mec* type and mortality is likely to be influenced by the proportion of community-acquired MRSA (CA-MRSA) and hospital-acquired MRSA (HA-MRSA) carrying specific SCC*mec* types during a given time. A previous study revealed that mortality rates of patients with CA-MRSA bacteremia were not significantly different compared to those of patients with MSSA bacteremia; however, HA-MRSA infections had a relatively high impact on mortality [27].

In the previous study from Japan on patients from 2014 to 2016, average values of length of stay and hospital charges were reported as 32.0 days and $15,762, respectively, in the MRSA patients group, while they were 28.7 days and $14,152, respectively, in the MSSA patients group [8]. These values were within the IQRs obtained in the univariate analysis in our study (Table 1). The estimated effects of MRSA compared to those of MSSA on length of stay and hospital charges in this study (Table 2) were also comparable (i.e., the confidence intervals almost overlapped) to those in a previous study from 2014 to 2016. On the other hand, concerning estimates of hospital charges of MRSA bacteremia, a previous meta-analysis showed that the estimated medical costs of MRSA bacteremia were $9,955 (95% CI: 8,952–10,957) higher than those of MSSA bacteremia [28]. The 95% CI in the previous meta-analysis was lower than the higher estimates observed in this study ($77,451 for MRSA bacteremia patients with HD or HF and $12,496 for those without HD or HF). The higher hospital charges for MRSA than those in previous studies may be a characteristic of university hospitals that provide advanced and specialized care, including cancer treatment. One of the primary reasons for the higher hospital charges in the MRSA infection group compared to those in the MSSA infection group is likely the significantly longer hospitalization period. Note that the duration of hospitalization was not included in the multiple regression model due to its strong association with the MRSA infection group to avoid issues of multicollinearity.

The hospital charges in the MRSA infection group were also higher than those in the MRSA colonization group. As mentioned in the previous paragraph, one of the primary reasons for this is likely the significantly longer duration of hospitalization in the MRSA infection group.

Regarding the hospital charges in the MRSA colonization group, even if a patient does not develop an infection but only colonization occurs, the hospital charges is as high as $15,952 (Table 3), which is higher than $12,525 for patients with MSSA infection (Table 1). We should keep in mind that patients with MRSA colonization do not necessarily indicate non-infectious conditions with any organisms other than MRSA, and should not be interpreted as a control group for non-infectious patients. Rather, the MRSA-colonized patients represent a diverse group, encompassing individuals with various symptoms and conditions. Indeed, in the MRSA colonization group, the percentage of patients requiring ICU admission and mechanical ventilation (Table 3) was as high as that in the MRSA-infected group. These results suggest that MRSA is likely to be detected in patients undergoing high levels of medical interventions. Specifically, in the MRSA colonization group, 52% of the patients had a history of ICU admission, while 50% of the patients with MRSA infection had a history of ICU admission. This could be attributed to the active surveillance conducted upon ICU admission, particularly undergoing surgery, to detect MRSA colonization. This surveillance is necessary to implement appropriate infection control measures, including isolation, in the ICU. Consequently, a high percentage of ICU admissions may be observed in patients colonized with MRSA, at least in the university hospital.

Regarding confounding factors, HD or HF was consistently identified and adjusted for in multiple regression analyses comparing MRSA and MSSA infections, as well as MRSA infection and MRSA colonization. While previous studies have indicated a poor prognosis for MRSA-infected patients undergoing HD or HF [10, 29], the findings of this study also suggest a high prevalence of HD or HF among patients infected with MRSA, thus resulting in increased hospitalization duration and elevated medical expenses.

This study has some limitations. First, the sample size (496 in-patients with MRSA or MSSA infections and 1,178 in-patients with MRSA infections or MRSA colonization) was small, and the data were obtained from a university hospital in Japan. Further studies are warranted to include additional data from other hospitals to explore the external validity of the study. Second, in the university hospital, not all hospitalized patients are tested for MRSA colonization. Although MRSA colonization is routinely monitored in the ICU ward, particularly for patients undergoing surgery, and NICU, other wards do not conduct routine colonization surveys, thereby resulting in a high percentage of ICU admissions among patients colonized with MRSA.

## Conclusions

This retrospective study compared the disease and economic burden between MRSA and MSSA infections and between MRSA infection and colonization. To the best of our knowledge, this is the first study to compare both the burden of disease and hospital charges between MRSA infection and colonization while adjusting for confounding factors. There is no evidence that MRSA infections have a higher mortality rate than MSSA infections or MRSA colonization do, thereby suggesting an improvement in the diagnosis and treatment process of MRSA infections in the university hospital. In contrast, MRSA infection was associated with longer length of stay and higher hospital charges than both MSSA infection and MRSA colonization. Furthermore, HD or HF was identified as a common underlying factor contributing to increased length of stay and hospital charges. The framework, which compares disease burden and hospital charges between infection and colonization based on the data integration and differentiation of the two groups, will be expanded to encompass other bacterial species of significant public health concern.

### Electronic supplementary material

Below is the link to the electronic supplementary material.

Supplementary Material 1

Supplementary Material 2

## Data Availability

Raw data files, including Japanese characters, are available upon request.

## Supplementary text

### MRSA bacteremia vs MSSA bacteremia

Comparison of clinical characteristics between matched patients with MRSA bacteremia and those with MSSA bacteremia (Table S6) revealed differences in the univariate analysis at a significance level of 0.1 for the following explanatory variables: C10_Diabetes without chronic complication (*P* = 0.096), ICU admission (*P* = 0.014), mechanical ventilation (*P* = 0.008), and HD or HF (*P* = 0.004). The explanatory variables were more prevalent in the MRSA bacteremia group, except for C10_Diabetes without chronic complications, than in the MSSA bacteremia group. There were also significant differences in length of stay (median, 57 vs. 31 days, *P* = 0.0003) and hospital charges (median, $42,982 vs. $13,013, *P* = 0.00004). However, there was no significant difference in the in-hospital mortality.

Next, multiple regression analyses, adjusted for confounding factors, were performed for length of stay and hospital charges, which showed a significant difference in the univariate analysis. For length of stay, confounding factors were ICU admission, mechanical ventilation, and HD or HF (Table S7). Based on the examination of pairwise associations among the confounding factors (Table S8), we selected HD or HF as a representative of those that were highly associated (*p* < 0.001) with each other. After adjustment, the effect of MRSA bacteremia compared to that of MSSA bacteremia on length of stay was estimated to be 1.41-fold (95% confidence interval [CI] 1.01–1.96, *P* = 0.044) (Table S9). For the total hospital charges, the confounding factors were ICU admission, mechanical ventilation, and HD or HF (Table S7). Based on the examination of pairwise associations among the confounding factors (Table S8), we selected HD or HF as representatives of those that were highly associated (*p* < 0.001) with each other. After adjustment, the effect of MRSA bacteremia compared to that of MSSA bacteremia was estimated to be 1.99-fold (95% CI 1.33–2.99), as shown in Table S9. According to the multiple regression model, the estimated increase in hospital charges for MRSA bacteremia patients with HD or HF compared to those for MSSA bacteremia patients with the same conditions was $77,451 (measured in dollars). The estimated increase in hospital charges for MRSA bacteremia patients without HD or HF compared to those for MSSA bacteremia patients with the same conditions was $12,496.

## Abbreviations

MRSA: Methicillin resistant Staphylococcus aureus
MSSA: Methicillin susceptible Staphylococcus aureus
JANIS: Japan Nosocomial Infections Surveillance
DPC: Diagnosis Procedure Combination
CL: Clinical laboratory
ARBI: Antimicrobial-resistant bacterial infection
ICU: Intensive care unit
NICU: Neonatal intensive care unit
HD: Hemodialysis
HF: Hemofiltration
CI: Confidence interval
IQR: Interquartile range
SCC*mec*: Staphylococcal cassette chromosome *mec*
CA-MRSA: Community-acquired MRSA
HA-MRSA: Hospital-acquired MRSA
