# Supplementary material for: Comparison of disease and economic burden between MRSA infection and MRSA colonization in a university hospital: a retrospective data integration study
Source: Antimicrob Resist Infect Control. 2024 Feb 29;13:27. doi: 10.1186/s13756-024-01383-8 (PMC10905874; doi:10.1186/s13756-024-01383-8)
Supplement: Supplementary file 2 — Supplementary Material 2 [file 13756_2024_1383_MOESM2_ESM.ppt]

## Slide 1
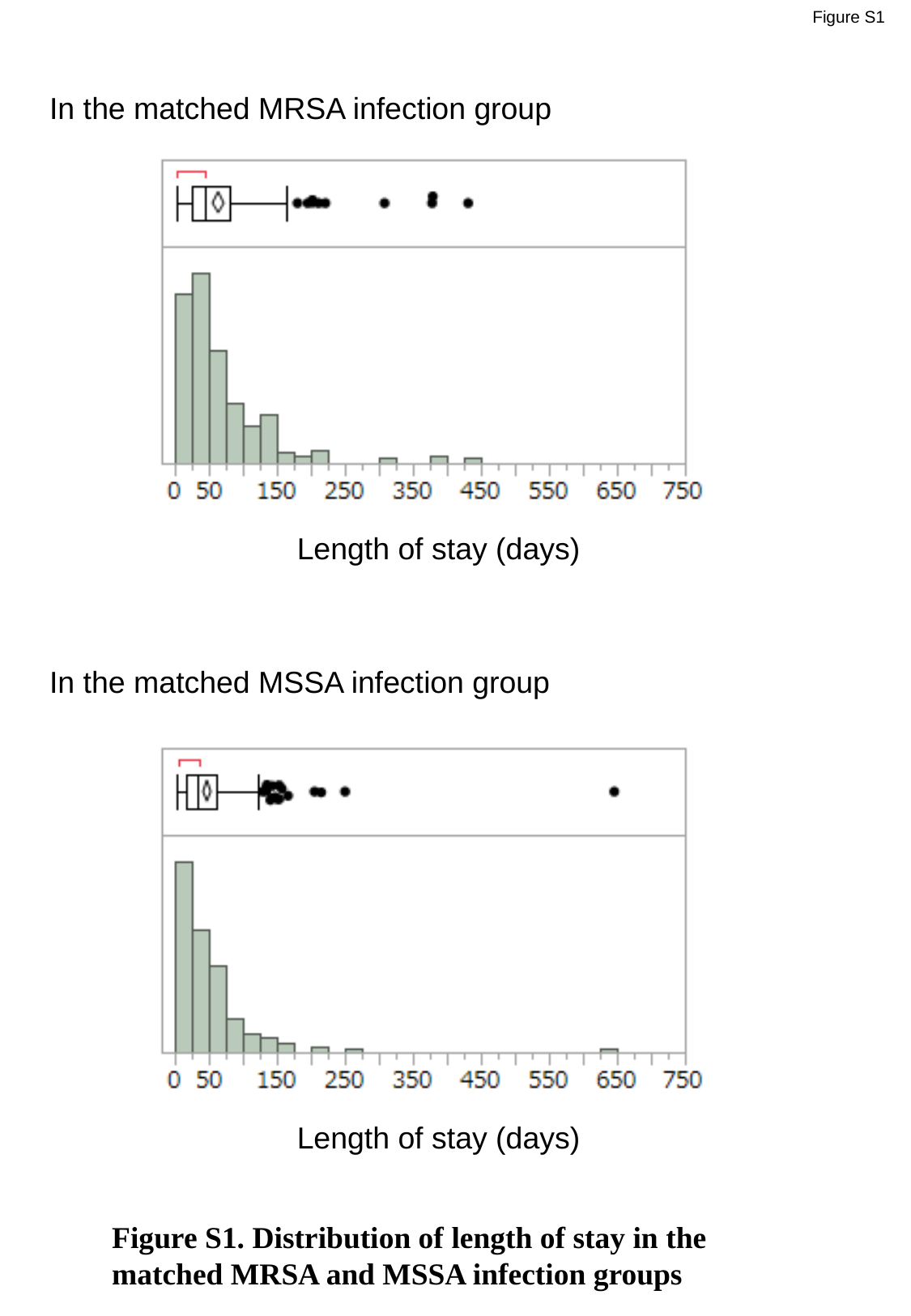

Figure S1
In the matched MRSA infection group
Length of stay (days)
In the matched MSSA infection group
Length of stay (days)
Figure S1. Distribution of length of stay in the
matched MRSA and MSSA infection groups

## Slide 2
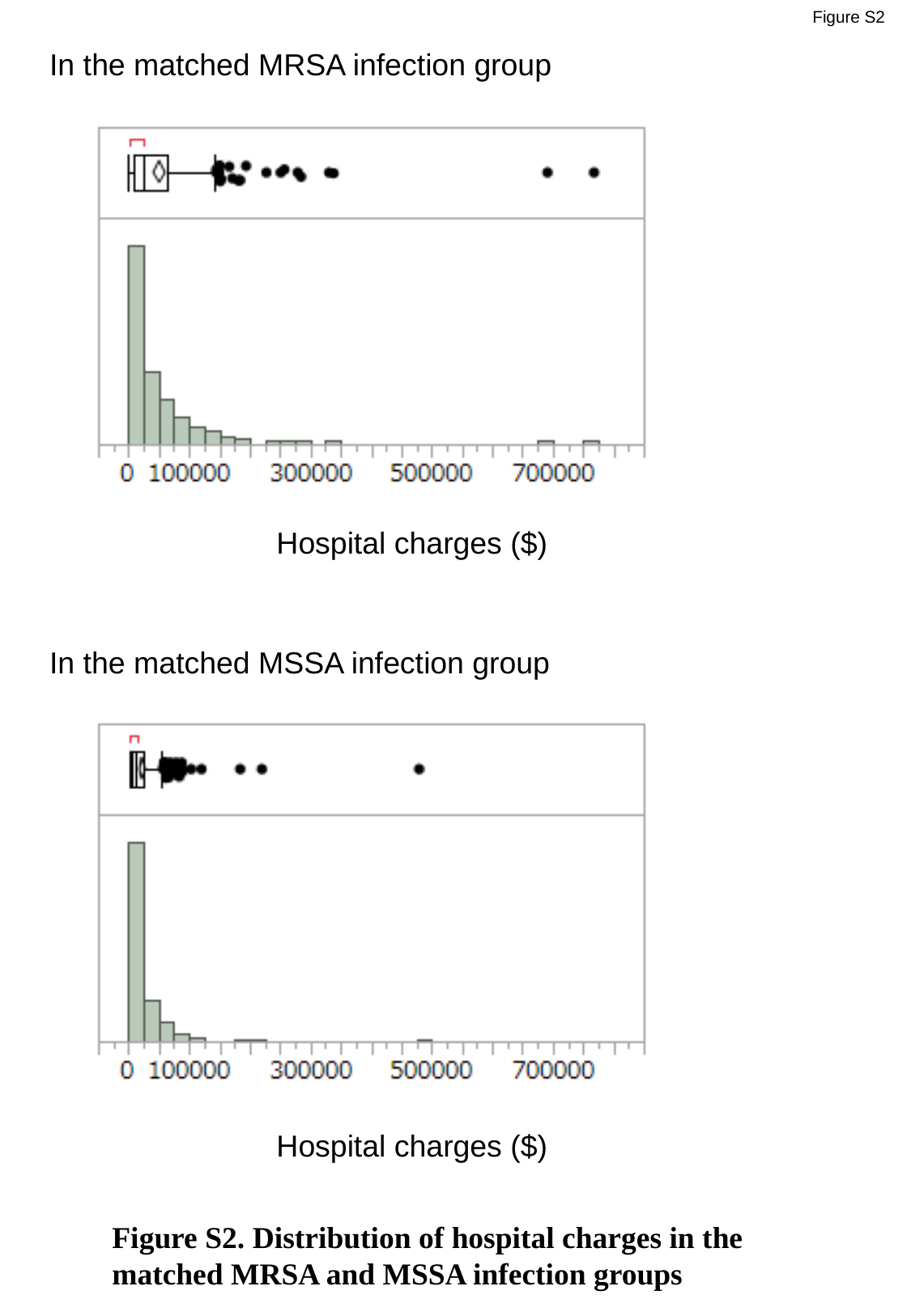

Figure S2
In the matched MRSA infection group
Hospital charges ($)
In the matched MSSA infection group
Hospital charges ($)
Figure S2. Distribution of hospital charges in the
matched MRSA and MSSA infection groups

## Slide 3
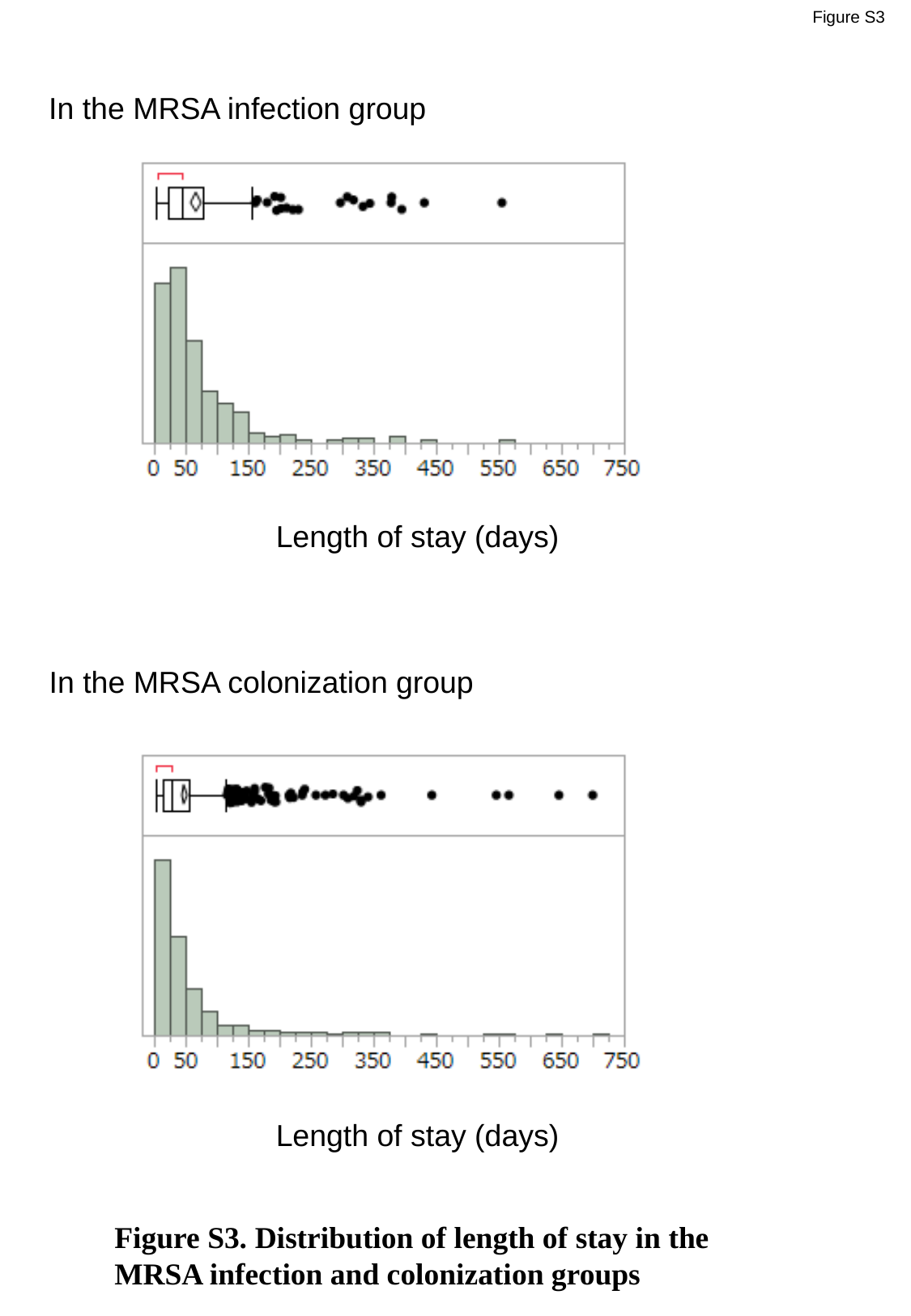

Figure S3
In the MRSA infection group
Length of stay (days)
In the MRSA colonization group
Length of stay (days)
Figure S3. Distribution of length of stay in the
MRSA infection and colonization groups

## Slide 4
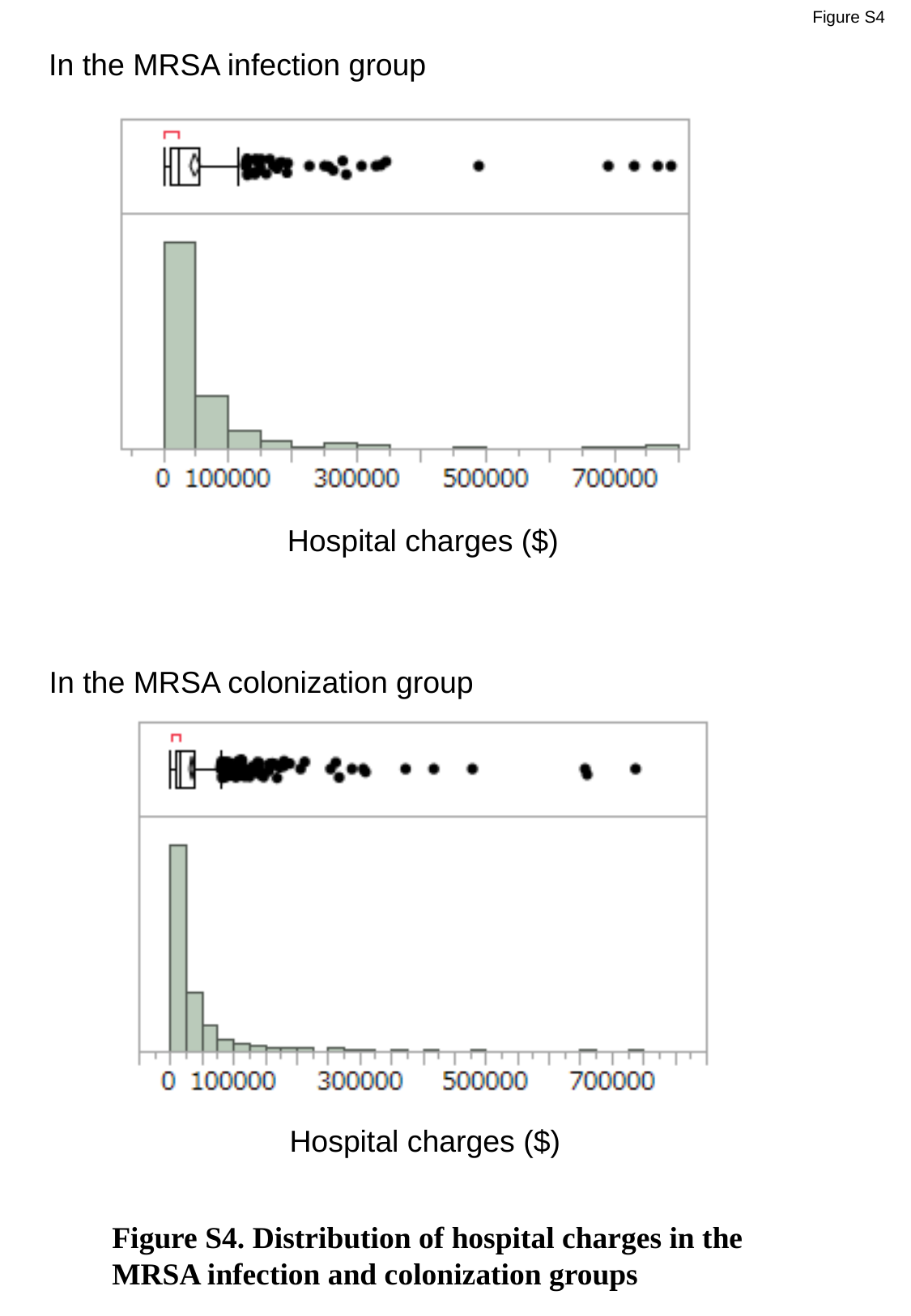

Figure S4
In the MRSA infection group
Hospital charges ($)
In the MRSA colonization group
Hospital charges ($)
Figure S4. Distribution of hospital charges in the
MRSA infection and colonization groups
